# Exploring metal artifact reduction using dual-energy CT with pre-metal and post-metal implant cadaver comparison: are implant specific protocols needed?

**DOI:** 10.1007/s00256-017-2750-2

**Published:** 2017-08-25

**Authors:** Ruud H. H. Wellenberg, Johanna C. E. Donders, Peter Kloen, Ludo F. M. Beenen, Roeland P. Kleipool, Mario Maas, Geert J. Streekstra

**Affiliations:** 1 Department of Radiology, AMC, Meibergdreef 9, 1105 AZ Amsterdam, The Netherlands; 2 Department of Orthopedic Surgery, AMC, Amsterdam, The Netherlands; 3 Orthopedic Trauma Service, Hospital for Special Surgery, New York, NY USA; 4 Department of Anatomy, AMC, Amsterdam, The Netherlands; 5 Department of Biomedical Engineering and Physics, AMC, Amsterdam, The Netherlands

**Keywords:** Dual-energy CT, Virtual monochromatic imaging, Metal artifacts, Orthopedic implants, Quantitative analysis

## Abstract

**Objective:**

To quantify and optimize metal artifact reduction using virtual monochromatic dual-energy CT for different metal implants compared to non-metal reference scans.

**Methods:**

Dual-energy CT scans of a pair of human cadaver limbs were acquired before and after implanting a titanium tibia plate, a stainless-steel tibia plate and a titanium intramedullary nail respectively. Virtual monochromatic images were analyzed from 70 to 190 keV. Region-of-interest (ROI), used to determine fluctuations and inaccuracies in CT numbers of soft tissues and bone, were placed in muscle, fat, cortical bone and intramedullary tibia canal.

**Results:**

The stainless-steel implant resulted in more pronounced metal artifacts compared to both titanium implants. CT number inaccuracies in 70 keV reference images were minimized at 130, 180 and 190 keV for the titanium tibia plate, stainless-steel tibia plate and titanium intramedullary nail respectively. Noise, measured as the standard deviation of pixels within a ROI, was minimized at 130, 150 and 140 keV for the titanium tibia plate, stainless-steel tibia plate and titanium intramedullary nail respectively.

**Conclusion:**

Tailoring dual-energy CT protocols using implant specific virtual monochromatic images minimizes fluctuations and inaccuracies in CT numbers in bone and soft tissues compared to non-metal reference scans.

## Introduction

Metal implants used for fracture fixation impede the diagnostic value of CT in the radiologic evaluation of fracture healing [1]. Especially when dealing with absent or delayed fracture healing it is of great importance to optimize CT image quality. Different metal alloys cause different grades of metal artifacts. In Europe, approximately 90% of the fixation plates, nails and screws are made of titanium where in the US, approximately 90% is made of stainless-steel (D. Höntzsch, personal communication, 2017). Stainless-steel results in more scatter and more pronounced artifacts in adjacent soft tissue and bone structures compared to titanium due to a higher molecular weight of stainless-steel [2]. Virtual monochromatic images, derived from dual-energy computed tomography (DECT) scans, are known to reduce metal artifacts by decreasing beam-hardening artifacts. Virtual monochromatic images at low keV show high overall image contrast and more pronounced artifacts whereas high keV images show reduced metal artifacts albeit with reduced overall image contrast. Several DECT studies found different optimal monochromatic energies focusing on metal artifact reduction (MAR) varying from 95–190 keV [2–10].

As different metal alloys result in different grades of artifacts it is likely that implant specific CT imaging protocols are needed to achieve optimal monochromatic energies (keV) for metal artifact reduction. As such, improved visualization of fracture healing and bone and soft tissue pathology may improve diagnosis, diagnostic accuracy and patient outcome. When determining optimal monochromatic energies for different metal alloys, it is essential to determine baseline measures for adjacent bone and soft tissue structures at different keVs without the presence of metal artifacts. In previous studies, no reference was used in either patient or cadaver studies focusing on metal artifact reduction due to the absence of non-metal CT data [5–7, 10–14]. In these reports, it is unclear how successful metal artifact corrections took place and if original non-metal baseline measures were reached.

The aim of this study was to quantify and optimize metal artifact reduction in relevant bone and soft tissue structures using virtual monochromatic dual-energy CT images in intramedullary and extramedullary fixation implants placed in the lower leg compared to non-metal reference scans.

## Methods

A pair of fresh frozen human cadaveric lower limbs from the body-donating program of our university, in accordance to Dutch law article 21 of the Burial and Cremation Act (BWBR0005009), was used. Dual-energy scans were acquired on a Siemens Somaton Force dual-source CT scanner (Siemens Healthcare, Forchheim, Germany) using 100-kVp (tube A) and tin filtered 150-kVp (tube B), 128 × 0.6 mm collimation, a pitch of 0.5, 1.5-mm slice thickness and 144 and 296 mAs for tube A and B, respectively. A sharp reconstruction filter (Qr59), also used in clinical practice, was chosen resulting in sharp edges, thereby enhancing the evaluation of osseous structures. After obtaining non-metal reference scans a titanium tibia plate, a stainless-steel tibia plate and a titanium intramedullary nail (DePuy/Synthes, Amersfoort, The Netherlands) were implanted respectively and repeated CT scans were obtained. Surgical procedures were performed under supervision of a fellowship-trained orthopedic trauma surgeon using standard approach and fixation techniques. Soft tissues were closed following placement of the fixation implants.

**Fig. 1:**
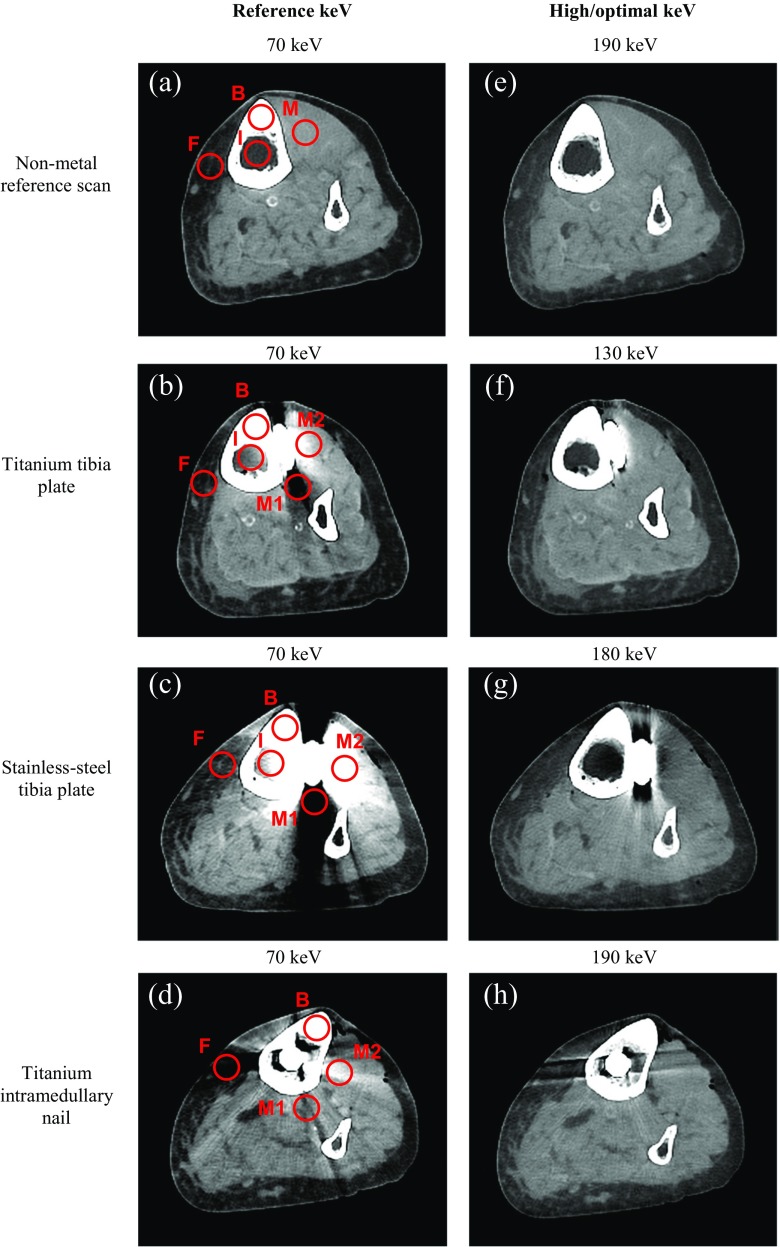
**a**–**d** Show 70 keV images with ROIs of the non-metal reference (**a**), titanium tibia plate (**b**), stainless-steel tibia plate (**c**) and the titanium intramedullary nail (**d**), respectively. **e** Shows a 190 keV image of the non-metal reference scan. **f**–**h** Show images at optimal keVs of 130, 180 and 190 keV for the implanted titanium tibia plate, stainless-steel tibia plate and the titanium intramedullary nail respectively. Virtual monochromatic images at high keV show reduced artifacts and lower overall image contrast compared to reference images at 70 keV. ROI measurements (illustrated *in red*) were performed in relevant bone and soft tissues: *B* bone, *M* muscle (*M1* = dark streak artifact, *M2* = bright streak artifact), *F* fat and *I* intramedullary cavity

Thin axial images were loaded on a SyngoVia workstation (version 3.0.0.16, Siemens Healthcare, Forchheim, Germany). For the titanium tibia plate, stainless-steel tibia plate, intramedullary nail and the reference scan, virtual monochromatic images were created from 70 to 190 keV, with steps of 10 keV, where 70 keV served as a reference. A reference of 70 keV was chosen since the average energy of a 120-kVp conventional CT scan is approximately 70 keV [15]. The left lower leg, containing the implants, was used for further analysis. For each setting the most suitable axial slices that not contained air and drill holes of previously implants screws were used for quantitative region of interest (ROI) measurements. Image J (version 1.48v, National Institute of Health, Bethesda, Maryland, USA) and Matlab (version 2014b, Natick, Massachusetts, USA) were used to create measurement templates for each of the four settings. Uniform circular ROIs with a diameter of 15 pixels were placed in the most affected and clinical relevant regions regarding muscle, fat, cortical bone and inside the intramedullary tibia canal where CT numbers and noise, measured as the standard deviation (SD) of pixels within a ROI, were measured (Fig. 1). In muscle, ‘M1’ was placed in a dark streak artifact region and ‘M2’ was placed in a bright streak artifact region with respectively decreased and increased CT numbers. Optimal keVs regarding CT number accuracy (|ΔCT number|) and SDs (|ΔSD|) were determined by searching for minimal absolute differences between the non-metal reference data and metal implant data.

## Results

### Non-metal

**Fig. 2:**
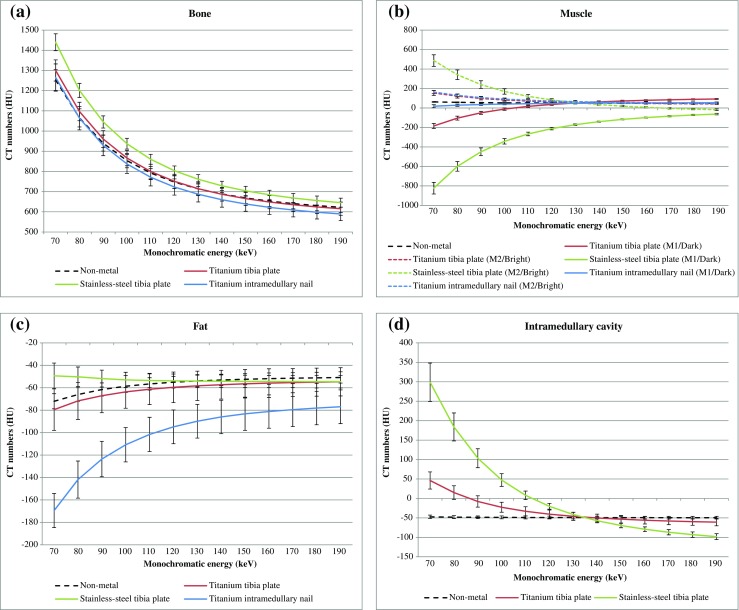
**a** CT numbers and standard deviations (SDs) of regions-of-interest (ROIs) placed in bone for each of the four scans: non-metal reference, titanium tibia plate, stainless-steel tibia plate and the titanium intramedullary nail are shown from *70* to *190* keV. Differences in CT numbers compared to non-metal reference results were largest for the stainless-steel tibia plate. CT number inaccuracies and standard deviations decreased at higher keV. **b** CT numbers and standard deviations (SDs) of regions-of-interest (ROIs) placed in muscle for each of the four scans: non-metal reference, titanium tibia plate, stainless-steel tibia plate and the titanium intramedullary nail are shown from *70* to *190* keV. In muscle, two ROIs were placed where M1 was placed in a dark streak artifact region and M2 was placed in a bright streak artifact region. Differences in CT numbers compared to non-metal reference results were largest for the stainless-steel tibia plate. CT number inaccuracies and standard deviations decreased at higher keV. **c** CT numbers and standard deviations (SDs) of regions-of-interest (ROIs) placed in fat for each of the four scans: non-metal reference, titanium tibia plate, stainless-steel tibia plate and the titanium intramedullary nail are shown from *70* to *190* keV. In this tissue type, despite the differences in CT numbers compared to non-metal reference results are small compared to bone and muscle. These differences were largest for titanium intramedullary nail. CT number inaccuracies and standard deviations decreased at higher keV. **d** CT numbers and standard deviations (SDs) of regions-of-interest (ROIs) placed in the intramedullary cavity for the non-metal reference, titanium tibia plate and stainless-steel tibia plate are shown from *70* to *190* keV. The CT number differences, measured in the intramedullary cavity, are largest for the stainless-steel tibia plate. CT number inaccuracies and standard deviations decreased at higher keV. An optimum can be observed between *130* and *140* keV for both implants

CT numbers and standard deviations (SD) varied within the range of 70–190 keV (Fig. 2, Table 1). The SDs in bone and soft tissues decreased from 70 to 190 keV, with the largest decrease in bone. In the non-metal scans overall image contrast decreased at high keVs. The contrast between muscle and fat at 190 keV decreased 27% compared to 70 keV. Positive CT numbers of bone and muscle decreased 50 and 28% while negative CT numbers of fat increased 29% at 190 keV compared to 70 keV results (Fig. 2).

**Table 1:** CT numbers and standard deviations of regions-of-interest placed in bone and soft tissues are given from *70 keV* up to *190 keV* with steps of 10 keV. Additionally, contrast between muscle and fat in non-metal reference scans, measured in absolute differences in CT numbers, was provided

	70 keV	80 keV	90 keV	100 keV	110 keV	120 keV	130 keV	140 keV	150 keV	160 keV	170 keV	180 keV	190 keV
Bone													
Non-metal	1,252 ± 52	1,065 ± 45	940 ± 39	854 ± 35	792 ± 33	747 ± 31	714 ± 29	688 ± 28	669 ± 27	653 ± 27	641 ± 26	631 ± 26	623 ± 25
Titanium tibia plate	1,300 ± 52	1,097 ± 46	960 ± 41	866 ± 38	800 ± 36	751 ± 35	714 ± 34^a^	687 ± 34	665 ± 33	648 ± 33	635 ± 33	624 ± 33	615 ± 33
Stainless-steel tibia plate	1,440 ± 42	1,201 ± 35	1045 ± 30	937 ± 27	860 ± 25	803 ± 24	761 ± 23	729 ± 23	704 ± 22	684 ± 23	669 ± 22	656 ± 22	646 ± 22^a^
Titanium intramedullary nail	1,265 ± 67	1,064 ± 58^a^	929 ± 51	837 ± 46	771 ± 43	723 ± 40	687 ± 38	660 ± 37	639 ± 36	622 ± 35	609 ± 34	598 ± 34	589 ± 33
Muscle													
Non-metal	62 ± 5	58 ± 4	55 ± 4	53 ± 4	52 ± 4	51 ± 3	50 ± 3	49 ± 4	49 ± 3	49 ± 3	48 ± 3	48 ± 3	48 ± 3
Titanium tibia plate (M1/dark)	−184 ± 43	−104 ± 29	−50 ± 20	−11 ± 14	16 ± 10	36 ± 7	51 ± 7^a^	63 ± 8	72 ± 10	79 ± 11	84 ± 12	89 ± 13	92 ± 14
Titanium tibia plate (M2/bright)	154 ± 25	122 ± 20	98 ± 15	82 ± 12	72 ± 10	64 ± 9	58 ± 8	53 ± 8	50 ± 7^a^	47 ± 7	45 ± 7	43 ± 7	42 ± 6
Stainless-steel tibia plate (M1/dark)	−824 ± 50	−600 ± 47	−450 ± 46	−342 ± 46	−267 ± 47	−212 ± 47	−172 ± 47	−141 ± 47	−117 ± 47	−99 ± 48	−84 ± 48	−72 ± 48	−63 ± 49^a^
Stainless-steel tibia plate (M2/bright)	487 ± 60	341 ± 49	240 ± 40	170 ± 28	120 ± 18	83 ± 12	56 ± 8^a^	35 ± 6	19 ± 6	7 ± 6	−3 ± 7	−11 ± 7	−18 ± 8
Titanium intramedullary nail (M1/dark)	18 ± 15	29 ± 11	36 ± 9	40 ± 8	44 ± 7	46 ± 7	48 ± 7	50 ± 7^a^	51 ± 7	52 ± 8	53 ± 8	53 ± 8	54 ± 8
Titanium intramedullary nail (M2/bright)	163 ± 17	130 ± 14	107 ± 11	91 ± 12	80 ± 12	72 ± 11	65 ± 12	61 ± 12	57 ± 12	54 ± 12	52 ± 12	50 ± 12	49 ± 12^a^
Fat													
Non-metal	−72 ± 7	−66 ± 6	−62 ± 6	−59 ± 6	−57 ± 5	−55 ± 5	−54 ± 5	−53 ± 5	−52 ± 5	−52 ± 5	−51 ± 5	−51 ± 5	−51 ± 5
Titanium tibia plate	−79 ± 19	−72 ± 17	−67 ± 15	−64 ± 15	−61 ± 14	−60 ± 14	−58 ± 13	−57 ± 13	−56 ± 13	−56 ± 13	−55 ± 12	−55 ± 12^a^	−55 ± 13
Stainless-steel tibia plate	−49 ± 11	−50 ± 9	−52 ± 8	−53 ± 7	−54 ± 6	−54 ± 6	−54 ± 6^a^	−54 ± 6	−54 ± 6	−54 ± 5	−54 ± 5	−55 ± 6	−55 ± 6
Titanium intramedullary nail	−169 ± 15	−142 ± 17	−124 ± 16	−111 ± 15	−102 ± 15	−95 ± 15	−90 ± 15	−86 ± 15	−83 ± 15	−81 ± 15	−80 ± 15	−78 ± 15	−77 ± 15^a^
Contrast between muscle-fat (non-metal)	135	124	117	112	108	106	104	103	101	101	100	99	99
Intramedullary cavity													
Non-metal	−47 ± 4	−48 ± 4	−48 ± 4	−49 ± 4	−49 ± 4	−49 ± 4	−49 ± 4	−49 ± 3	−49 ± 3	−49 ± 3	−49 ± 3	−49 ± 3	−49 ± 3
Titanium tibia plate	46 ± 22	15 ± 17	−8 ± 15	−23 ± 13	−33 ± 12	−40 ± 11	−46 ± 10	−50 ± 10^a^	−53 ± 10	−56 ± 10	−58 ± 9	−60 ± 9	−61 ± 9
Stainless-steel tibia plate	299 ± 49	184 ± 36	103 ± 24	47 ± 16	8 ± 11	−20 ± 7	−41 ± 6^a^	−57 ± 5	−70 ± 6	−79 ± 6	−87 ± 7	−93 ± 7	−98 ± 8

^a^Minimal CT number differences compared to non-metal reference CT numbers

### Metal

**Fig. 3:**
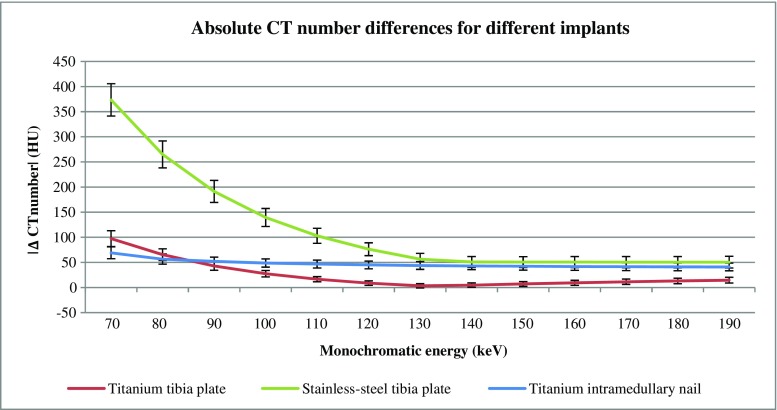
Absolute differences in CT numbers for the three different metal implants compared to non-metal results are shown. Quantitative results of all ROIs were averaged in order to obtain optimal keVs for each implant. CT number differences compared to non-metal results were smallest at *130*, *180* and *190* keV for the titanium tibia plate, stainless-steel tibia plate and titanium intramedullary nail respectively

The stainless-steel implant resulted in the most severe metal artifacts whereas the titanium intramedullary nail resulted in the least severe metal artifacts based on quantitative measurements (Figs. 1, 2 and 3; Table 1). CT numbers in 70 keV images were decreased in dark streak artifact regions and increased in bright streak artifact regions. We estimated the keVs where the measured CT numbers in metal scans were similar to those of the non-metal reference scans. For bone, CT numbers of non-metal results were reached for the titanium tibia plate and intramedullary nail at 130/140 keV and 80 keV respectively. In case of the stainless-steel implant, CT numbers of non-metal scans were not reached (Fig. 2a; Table 1). Also in ROIs placed in muscle, fat and the intramedullary cavity reference values were not always reached at higher keVs. When CT numbers of non-metal scans were reached for ROIs placed in muscle, fat and the intramedullary cavity, this was accomplished between 130 and 150 keV (Fig. 2b–d; Table 1). When reference values were not reached, differences in CT numbers compared to reference values were minimal at 190 keV, with a maximal difference of 101 HU for ‘M1’ in case of the stainless-steel tibia plate (Fig. 2b; Table 1).

Optimal keVs differ for bone and soft tissues. In order to generate results for specific implants and to obtain optimal keVs, CT number and SD inaccuracies were averaged for each implant and compared to non-metal reference data (Fig. 3). The least CT number inaccuracies of the relevant structures in 70 keV images were observed at 130, 180 and 190 keV for the titanium tibia plate, stainless-steel tibia plate and titanium intramedullary nail respectively. At these optimal keVs the CT number inaccuracies were decreased with 96, 87 and 73% for the titanium tibia plate, stainless-steel tibia plate and titanium intramedullary nail respectively (Fig. 3). Standard deviations were increased compared to the reference scan for all ROIs due to the influence of metal artifacts. Those metal artifacts decreased for nearly all ROIs at higher keVs resulting in lower SDs. SDs were minimized at 130, 150 and 140 keV for the titanium tibia plate, stainless-steel tibia plate and titanium intramedullary nail respectively. In these images SDs decreased with 73, 66 and 35% for the titanium tibia plate, stainless-steel tibia plate and titanium intramedullary nail respectively (Fig. 3).

## Discussion

In this study, we found that virtual monochromatic images computed from dual-energy CT scans reduced metal artifacts by reducing CT number inaccuracies and fluctuations in bone and soft tissues. These inaccuracies and fluctuations were highest at the reference monochromatic energy of 70 keV and decreased at higher keVs. The stainless-steel alloy resulted in more severe artifacts at equal keVs compared to both titanium implants, which was expected due to a higher atomic weight for the stainless-steel implant. Optimal monochromatic energies, based upon CT number analysis, tuned out to be dependent on the type of metal implant. These optimal energies differ for the titanium tibia plate, stainless-steel tibia plate and titanium intramedullary nail (130, 180 and 190 keV respectively).

In our study, we used non-metal CT data to determine baseline measures unlike in patient studies where real non-metal reference cannot be used [5–7, 10–14]. Only in some phantom studies non-metal scans were taken into account and were used as a reference [2, 16]. Although phantom studies may generate valuable information about the effectiveness of metal artifact reduction methods, they do not reflect the situation of real anatomical structures. Han et al. 2014 did investigate non-metal scans; however, they made a comparison between two groups (patients with and without a metal hip prosthesis) instead of an ideal comparison within individual patients [17], which may underestimate statistical significance between CT values due to variability of CT values within a population. Most of the patient studies used internal references distal to the implant [5–7, 10, 11, 14]; however, these regions may also be affected by metal artifacts. In our approach, the use of standardized measurement templates on fixed locations over the range of 70–190 keV enhanced the reliability of the measurements, including those of reference CT values without metal.

Few studies focus on the evaluation of bone and soft tissue structures regarding fixation implants. Some studies focus on pedicles and screws [4, 7, 8, 11] and others on larger hip implants [3, 6, 12, 14, 17] or dental implants [12, 18]. Filograna et al. (2015) investigated various metallic orthopedic implants in post-mortem studies, including fixation implants in the extremities. They found similar results where streak intensities decreased with an optimal keV between 130–148 keV [9]. Zhou et al. (2011) also investigated internal and external fixation implants in the upper and lower extremities with an optimal keV of 130 keV [13]. Bamberg et al. (2011) found optimal keVs of 95–150 keV, including results of nine patients with fixation implants [10].. Again, none of these clinical studies or post-mortem studies used non-metal reference scans.

The influence of different metal alloys and effectiveness of DECT in metal artifact reduction is investigated in some studies. Like in our study, these studies found that titanium implants result in less severe metal artifacts compared to metals with a higher atomic weight such as aluminum, stainless-steel, cerrobend and cobalt-chrome [2, 5, 8, 14, 18]. Lee et al. (2012) found optimal keVs of 80 and 110 keV for titanium and stai nless steel fixation plates respectively based on phantom results [5]. Komlosi et al. (2015) found optimal keVs of 90 keV and higher and 110 keV and higher for respectively titanium and cobalt-chrome cervical spine implants [8]. Meinel et al. (2012) investigated titanium and steel hip prostheses and found that steel hip prosthesis result in more severe artifacts; however an alloy specific optimal keV was not provided and they stated that a keV of 113 keV ranging from 100–130 keV is optimal [14].

## Limitations and conclusions

This study has limitations. We only performed quantitative analyses to determine optimal keVs and did not include subjective image quality scoring by radiologists. A clinical study including subjective image quality scoring by multiple observers focusing on metal artifact reduction, overall image quality and diagnostic accuracy in detecting bone and soft tissue pathology will provide valuable insides in the diagnostic value of virtual monochromatic images for different implants in patients.

Combining DECT with additional metal artifact reduction software may further improve image quality due to more effective metal artifact reduction by reducing photon-starvation and scatter artifacts [2, 5, 12, 17, 18]. However, combining MAR software with dual-energy CT imaging is currently not yet available on this system; furthermore when using MAR software, despite the fact that overall image contrast is not degraded compared to high keV virtual monochromatic results, new artifacts may be introduced [2, 17].

Choosing the optimal keV for each patient individually is both challenging and time consuming. When choosing a keV that is too low, metal artifacts will be more pronounced resulting in an overall decreased image quality compromising the evaluation of bone and soft tissue structures. On the other hand, when choosing a keV that is too high, overall image contrast will be decreased which impedes the diagnostic quality. We observed that most of the artifacts were reduced between 70–130 keV. In case of heavier metals, implants with a more complex geometry and in case of bilateral implants, a higher keV can be chosen since some metal artifact reduction is observed above 130 keV. Therefore, we suggest that the use of implant specific protocols is more beneficial than using generalized protocols for all implants. In a clinical setting, radiological evaluation using 70 keV images and implant specific optimal virtual monochromatic images with minimal CT number inaccuracies and fluctuation is therefore advised.
